# Supplementary material for: Development of chemokine network inhibitors using combinatorial saturation mutagenesis
Source: Commun Biol. 2025 Apr 3;8:549. doi: 10.1038/s42003-025-07778-6 (PMC11969024; doi:10.1038/s42003-025-07778-6)
Supplement: Supplementary file 4 — Supplementary Data 2 [file 42003_2025_7778_MOESM4_ESM.zip › SupplementaryData3/scripts/xlogo.tar!/./script/analysis/JK/JK_HD2XC_highest_XC_minCC_logo.pdf]

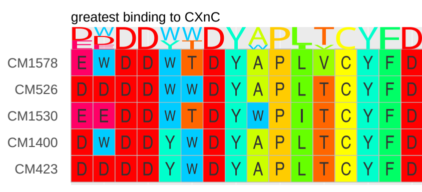

greatest binding to CXnC
CM1578 E W D D W T D Y A P L V C Y F D
CM526 D D D D W W D Y A P L T C Y F D
CM1530 E E D D W T D Y W P I T C Y F D
CM1400 D W D D Y W D Y A P L T C Y F D
CM423 D D D D Y W D Y A P L T C Y F D
